# Dielectrophoretic Separation of Live and Dead Monocytes Using 3D Carbon-Electrodes

**DOI:** 10.3390/s17112691

**Published:** 2017-11-22

**Authors:** Yagmur Yildizhan, Nurdan Erdem, Monsur Islam, Rodrigo Martinez-Duarte, Meltem Elitas

**Affiliations:** 1 Faculty of Engineering and Natural Sciences, Sabanci University, Istanbul 34956, Turkey; yildizhan@sabanciuniv.edu (Y.Y.); nurdanerdem@sabanciuniv.edu (N.E.); 2 Mechanical Engineering Department, Clemson University, Clemson, SC 29631, USA; monsuri@g.clemson.edu (M.I.); rodrigm@clemson.edu (R.M.-D.)

**Keywords:** dielectrophoresis, cell separation, carbon-electrode, microfluidics, BioMEMS (biomedical microelectromechanical systems)

## Abstract

Blood has been the most reliable body fluid commonly used for the diagnosis of diseases. Although there have been promising investigations for the development of novel lab-on-a-chip devices to utilize other body fluids such as urine and sweat samples in diagnosis, their stability remains a problem that limits the reliability and accuracy of readouts. Hence, accurate and quantitative separation and characterization of blood cells are still crucial. The first step in achieving high-resolution characteristics for specific cell subpopulations from the whole blood is the isolation of pure cell populations from a mixture of cell suspensions. Second, live cells need to be purified from dead cells; otherwise, dead cells might introduce biases in the measurements. In addition, the separation and characterization methods being used must preserve the genetic and phenotypic properties of the cells. Among the characterization and separation approaches, dielectrophoresis (DEP) is one of the oldest and most efficient label-free quantification methods, which directly purifies and characterizes cells using their intrinsic, physical properties. In this study, we present the dielectrophoretic separation and characterization of live and dead monocytes using 3D carbon-electrodes. Our approach successfully removed the dead monocytes while preserving the viability of the live monocytes. Therefore, when blood analyses and disease diagnosis are performed with enriched, live monocyte populations, this approach will reduce the dead-cell contamination risk and achieve more reliable and accurate test results.

## 1. Introduction

Precision in medicine widely relies on the measurements obtained from the body fluids such as blood, urine and sweat samples. Precise, quantitative and high-quality characteristics from these diagnostic measurements demand reliable, repeatable and accurate sample preparation. Recently developed diagnostic tools and biosensors are able to prepare their own samples and use these samples serially to run the bioassays, as distinct from the introduction of separately prepared specimens. Performing both sample preparation and analysis on the same device provides not only a constant micro-environment but also reduces experimental errors occurring on different scales using traditional batch culture assays. Furthermore, the methods used to isolate or characterize biological samples need to preserve the genotype and phenotype of the cells to resemble the status of the diseases.

In the literature, several methods ranging from density-based approaches to antibody-mediated recognition assays have been reported to separate and purify specific cell populations from whole body fluids [1,2,3,4,5,6,7,8,9,10,11,12]. Since most density-based methods are based on centrifugation, these purification methods are more valuable at the population-level than at the single-cell level [8,9,10,11,12,13]. Fluorescent-activated (FACS) and magnetic-activated (MACS) cell sorting methods are among the most common cell-separation methods, based on antibody-binding assays [14,15,16,17]. In these assays, cells are conjugated with antibodies either attached to a fluorescent dye or a charged particle to be identified in FACS or MACS, respectively. However, cell labeling might change the phenotype of some cells and might cause biases in their characterization, especially for immunological cells, which rapidly responds and adapts to environmental changes [18,19].

As an alternative to FACS and MACS, researchers have often investigated the dielectrophoretic identification and separation methods (DEP) since Pohl introduced this phenomenon in the 1950s [20]. The main reasons that have made DEP one of the long lasting cell characterization and separation methods are: (i) DEP preserves the viability, culturability, genetic and phenotypic properties of the cells [21]; (ii) DEP employs the intrinsic dielectric properties of the cells [22]; (iii) DEP is contact-free and low cost [23,24,25,26,27,28]; (iv) DEP provides high-yield for the downstream analysis of the cells [29].

In the literature, the majority of the DEP work has been reported regarding the utilization of DEP in the isolation of viable cells from nonviable ones [20,30,31,32,33], yet there remain critical issues to be addressed and improvements to be made [34]. For example, cell clogging might be a problem when reservoir-based dielectrophoresis has been applied to trap cells selectively [35]. Sometimes, a high-conductive DEP buffer can cause electrolysis problems that form bubbles and dissolve the metal electrodes [36,37]. The contactless dielectrophoresis applied by Shafiee and his group succeeded in isolating live leukemia cells from dead ones in a highly efficient way without dead-cell contamination. However, the live cells lysed due to the applied high electrical field, which had created DEP forces [38].

The 3D carbon-DEP method combines the advantages of both metal- and isolator-based DEP (iDEP) approaches and provides solutions to the problems related to metal-electrode based and iDEP-based devices. First of all, the fabrication of the 3D carbon-electrodes is based on the pyrolysis of a negative epoxy-based UV photoresist, SU-8. Therefore, its fabrication is relatively less complicated and inexpensive in comparison to fabrication of 3D metal-electrodes. Second, in order to generate an electric field, 3D carbon-electrodes require only tens of volts, whereas iDEP requires as high as hundreds or thousands of volts. Third, 3D carbon-electrodes minimize the sample electrolysis thanks to the wider electrochemical stability of carbon. Finally, carbon-electrodes have excellent biocompatibility and mechanical properties, as reported in [39]. Since the height of the electrodes is taller in 3D carbon-DEP chips, the inner volume of the DEP chip can be used more efficiently to generate high-throughput [40,41]. The advantages of 3D carbon-DEP chips have been reported using various sample types such as particles [40], yeast cells [41], bacteria [42] and DNA [43].

Here, we applied 3D carbon-dielectrophoretic investigations for monocytes. Among the blood cells, monocytes are the largest leukocyte population, composing nearly 2–10% of the white blood cells. They are associated with certain inflammatory diseases such as atherosclerosis [44] and bowel disease [45,46]. They support the immune system against several viral, fungal or bacterial infections [47,48,49,50,51,52,53]. Moreover, an increased and decreased monocyte number can be a symptom of particular diseases such as tuberculosis [54,55], cancer [56], and Chronic Myelomonocytic Leukemia (CMML) that originates in bone marrow, resulting in high levels of monocyte numbers in the blood [57]. Although their irregular shape and kidney-shaped nuclei distinguish them from other types of white blood cells, their morphology-based separation is not adequate to perform clinical assays due to their extensive heterogeneity [58]. Therefore, dielectrophoretic separation and characterization methods for monocytes emerged.

In this study, we applied the 3D carbon-DEP method for the characterization of live and dead U937 (human myeloid leukaemia) monocyte cells obtained from the same cell culture. Next, we removed the dead monocytes from the live monocyte cells. We intended to enrich viable U937 monocytes without altering their genetic or phenotypic properties while reducing dead-cell contamination from the population in order to achieve accurate and reliable readouts from biological assays and clinical tests.

## 2. Theory

The dielectrophoretic force occurs when a dielectric particle is placed under an inhomogeneous electric field. It affects the particles either in an attractive or repulsive manner based on the permittivity of the particle and its surrounding medium [59,60], as formulated in Equation (1):

(1) $$F_{DEP}=2\pi \varepsilon_{m}r^{3}Re\left[\frac{\varepsilon_{eff}^{*}-\varepsilon_{m}^{*}}{\varepsilon_{eff}^{*}+2\varepsilon_{m}^{*}}\right]\Delta E_{rms}^{2}.$$

**Figure 1:**
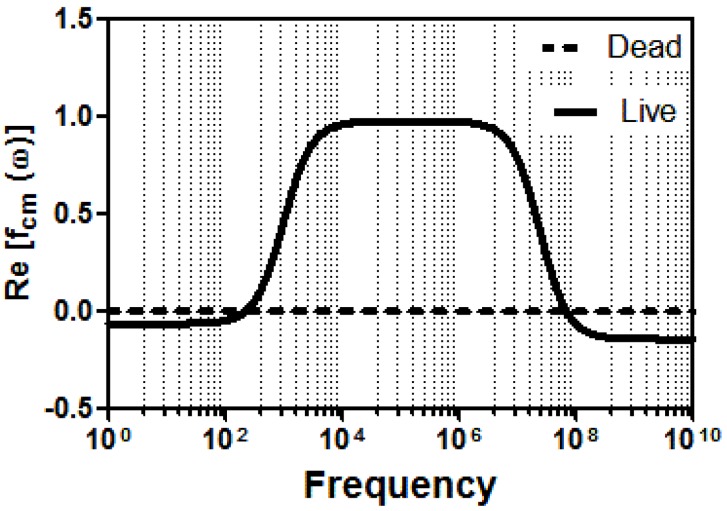
Characterization of the live and dead U937 monocytes. The $Re[f_{cm}\left(w\right)]$ vs. frequency (Hz) data is obtained using the dielectric properties of the live (continuous line) and dead (dashed line) monocytes.

More explicitly, the DEP force depends on the permittivity of the suspending medium ($\varepsilon_{m}$), the radius of the cell (*r*), the real part of the Clausius–Mossotti factor ($f_{cm}$) and the applied non-uniform electric field (*E*). The real part of the Clausius–Mossotti factor ($f_{cm}$) is calculated as in Equation (2):

(2) $$f_{cm}\left(w\right)=\frac{\varepsilon_{eff}^{*}-\varepsilon_{m}^{*}}{\varepsilon_{eff}^{*}+2\varepsilon_{m}^{*}},$$

where the $\varepsilon_{m}^{*}$ is being the complex permittivity of the medium and $\varepsilon_{eff}^{*}$ is the effective complex permittivity of the cell, given in Equation (3), based on the single shell model [61,62]:

(3) $$\varepsilon_{eff}^{*}=\varepsilon_{mem}^{*}\frac{{\left(\frac{r}{r-d}\right)}^{3}+2\frac{\varepsilon_{int}^{*}-\varepsilon_{mem}^{*}}{\varepsilon_{int}^{*}+2\varepsilon_{mem}^{*}}}{{\left(\frac{r}{r-d}\right)}^{3}-\frac{\varepsilon_{int}^{*}-\varepsilon_{mem}^{*}}{\varepsilon_{int}^{*}+2\varepsilon_{mem}^{*}}},$$

where the thickness of the cellular membrane (*d*), with complex permittivity ($\varepsilon_{mem}^{*}$), and the complex permittivity of the cytoplasm ($\varepsilon_{int}^{*}$) define the characteristic dielectric properties of the cells. When the cells are attracted towards the regions of the high electric field, the real part of the $f_{cm}$ has become positive, and the positive DEP (pDEP) has acted. When the cells are repelled from the regions of the high electric field, the negative DEP (nDEP) has occurred and the sign of the real part of the $f_{cm}$ has become negative. The transition from “nDEP to pDEP” or “pDEP to nDEP” appears at a specific frequency, which is called the “crossover frequency (CF)”. It has been widely used as a dielectric characteristic for the cells. It defines intrinsic cellular properties. For example, the dielectrophoretic response of a living cell is radically different from a dead one because the dead cell with an impaired membrane polarizes differently under the applied electric field [63,64]. Therefore, using the polarizability difference, viable cells are being purified from nonviable cells. For the numerical confirmation, the behavior of live and dead U937 monocytes was simulated using Matlab script (Version R2016a, The MathWorks Inc., Natick, MA, USA, see Appendix). The parameters for the numerical analysis are taken from [65]. Figure 1 shows the simulation results for the characteristic dielectrophoretic responses of the viable and nonviable U937 monocytes.

In this simulation, dielectrophoretic responses of live and dead U937 monocytes were scanned for the frequency ranging from 1–10${}^{10}$ Hz. Both cell types had 7 nm membrane thickness and $12.5\varepsilon_{0}$ membrane permittivity. Cell diameters were measured as 23 μm and 22 μm for the live and dead cells, respectively (see Supplementary Materials, Figure S1). For the live cells, the cytoplasm conductivity was taken as 0.5 S/m, cytoplasm permittivity $50\varepsilon_{0}$ and membrane conductivity as 10${}^{-6}$ S/m. For the dead cells, the cytoplasm conductivity was taken as 0.002 S/m, cytoplasm permittivity as $80\varepsilon_{0}$ and membrane conductivity as 0.01 S/m, since it was reported that membrane conductivity increases by $10^{4}$ fold when a cell is dead [31]. The surrounding medium for the cells had 0.002 S/m conductivity with the $80\varepsilon_{0}$ medium permittivity, where $\varepsilon_{0}:8.85$× 10${}^{-12}$ F/m.

## 3. Materials and Methods

### 3.1. 3D Carbon-DEP Device Fabrication

Fabrication of 3D glass-like carbon-microelectrodes has been explained in detail [40,41,42,43,66]. In brief, two-step photolithography process of SU-8 (Gersteltec, Pully, Switzerland), a negative-tone photoresist, on a silicon wafer was applied to create the 3D structure of the microelectrodes. Microelectrodes were then carbonized by heat treatment to 1000 ${}^{\circ }$C in a nitrogen atmosphere. The 3D carbon-DEP chip has 218 intercalated rows of 14 or 15 electrodes for a total of 3161 electrodes with 100 μm height and 50 μm diameter. Next, a thin layer of SU-8 was applied to insulate the planar connecting leads and to make the bottom channel planar. A 1.8 mm-wide, 3.2 cm-long channel was cut from a 127 μm-thick double-sided pressure sensitive adhesive (PSA, Switchmark 212R, Flexcon, Spencer, MA, USA) and adhered to a previously drilled polycarbonate. This arrangement was then manually positioned around the carbon-electrode array and sealed using a rolling press.

### 3.2. Low Conductive DEP Buffer Preparation

The low conductive DEP buffer was prepared via diluting 8.6% sucrose (Item no: LC-4469.1, neoFroxx, Hesse, Germany), 0.3% glucose (CAS Number 59-99-7, Sigma-Aldrich, Darmstadt, Germany) and 0.1% Bovine Serum Albumin (BSA, Product Code: P06-1391050, PAN-Biotech, Aidenbach, Germany) in distilled water [40]. The conductivity of the final suspension was 20 μS/cm, measured by a conductivity meter (Corning Model 311 Portable Conductivity Meter, Cambridge Scientific Products, Watertown, MA, USA).

### 3.3. Cell Preparation

The U937 monocyte cells are the human myeloid leukemia cell line, obtained from American Type Culture Collection (ATCC, Manassas, VA, USA) company. We cultured them in RPMI (Roswell Park Memorial Institute) - 1640 medium (Product Number: P04-18047, PAN-Biotech, Aidenbach, Germany) supplemented with 10% fetal bovine serum (FBS; PAN-Biotech, Aidenbach, Germany) in vented plastic flasks under a 5% CO${}_{2}$–95% air atmosphere in a humidified incubator at 37 ${}^{\circ }$C (EC 160 CO2 Incubator, Nuve, Ankara, Turkey). The live and dead monocytes were obtained from the same tissue culture flasks to eliminate culture to culture variation when they are grown in different flasks.

The cells were spun down at 3000 rpm (Z601039 - Hettich® EBA 20 centrifuge, MERCK, Darmstadt, Germany) for 5 min to remove any residual culture media and resuspended in DEP buffer twice. The cell number was determined using a haemacytometer (Catalog No: 0680030, Marienfeld-Superior, Lauda-Königshofen, Germany). Both for the characterization and separation of the live and dead monocytes, 3 × 10${}^{5}$ cells/mL were introduced into the DEP chip. The live and dead cells were distinguished using the Trypan blue dye (Sigma-Aldrich, Darmstadt, Germany). Viability of the cells was determined both in the low conductive DEP buffer and when the electric field is applied (Supplementary Materials Figures S2 and S3).

### 3.4. Experimental Setup

**Figure 2:**
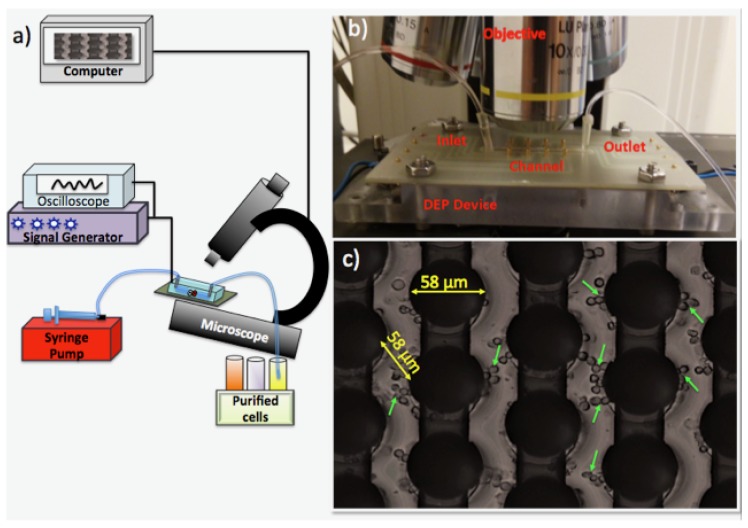
The experimental setup (**a**) equipment is used for the 3D carbon-Dielectrophoresis experiments include an upright microscope, an oscilloscope, a signal generator, a syringe pump and the 3D carbon-DEP chip; (**b**) the 3D carbon-DEP chip under the objectives; (**c**) the carbon-electrode array [43]. The green arrows show the monocytes.

Our experimental setup, presented in Figure 2, consists of a signal generator to create the electric field (Model: GFG-8216A, GW Instek, New Taipei City, Taiwan), an oscilloscope (Part Number: 54622D, Agilent Technologies, Santa Clara, CA, USA) to measure the amplitude and the frequency of the applied signal, a tabletop, upright microscope (Model: Nikon ME600 Eclipse, Nikon Instruments Inc., Melville, NY, USA) to monitor the cells, a computer to save and analyze the acquired images (Hewlett-Packard Company, Palo Alto, CA, USA), a programmable syringe pump (Model: NE-1000, New Era Pump Systems Inc, Farmingdale, NY, USA) to flow the cells and the DEP buffer , and our 3D carbon-DEP device. We inserted two 20–200 μL pipette tips (Manufacturer ID: 3120000917, Eppendorf, Hamburg, Germany) at the inlet and outlet of the microchannel to create reservoirs. The tygon microbore tubing (Manufacturer ID: AAQ02103-CP S-54-HL, Cole-Parmer, Vernon Hills, IL, USA) was used to connect the syringe and the microchannels of the 3D carbon-DEP chip.

### 3.5. Experimental Procedure

First, the system was sterilized via flowing 70% Ethanol and then DI (deionized) water [21]. Next, the 3D carbon-DEP chip was filled with the DEP buffer, and all the bubbles from the chip were removed. Afterwards, the cells were prepared as explained above and 40 μL cell suspension was loaded into the chip using a 10 μL/min flow rate. When cells reached to the region of the carbon electrodes, the flow was stopped and the cells were settled for 30 s. The signal with 20 Vpp (peak-to-peak voltage) and 50 kHz–1 MHz was applied for the cells in the 3D carbon-DEP device using the function generator [40]. When the electric field was 20 Vpp–300 kHz, the viable U937 monocytes exhibited pDEP while nonviable cells remained unresponsive. Thanks to the selective DEP forces, the live cells were trapped at the high-electric-field regions. Using the 1 μL/min flow rate, the drag force discarded the dead cells from the 3D carbon-DEP chip. The dead cells were also collected inside a collection tube for further confirmation. When all the dead cells were removed, the electric field was turned off, and the live cells were flown and collected for additional viability tests. The whole experiment was performed in 40 min.

### 3.6. Downstream Analysis

Both to enumerate cell number and confirm the viability of the collected cells, the cells inside the collection tubes were stained with Trypan blue and counted using the hemocytometer. The percentage of enriched live cells and removed dead cells were calculated normalizing the collected cell numbers to the initial cell number of the cell suspension, which is introduced into the 3D carbon-DEP chip and composed of live and dead monocytes.

### 3.7. Image Acquisition and Analysis

**Figure 3:**
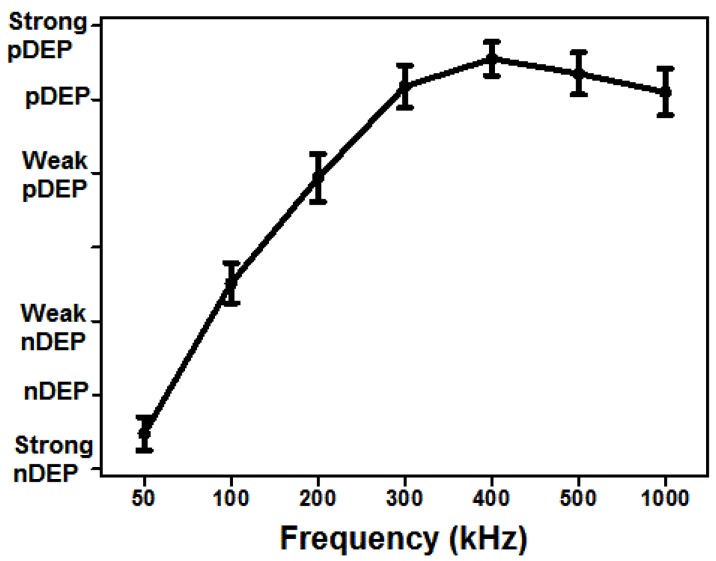
Dielectrophoretic behavior of the viable U937 monocytes. The electrode region was aligned with a grid that shows the strong nDEP (−3), nDEP (−2), weak nDEP (−1), crossover (0), weak pDEP (1), pDEP (2) and strong pDEP (3) behaviors of the cells. The mean values with the standard deviations represent 80 cells.

All the images were captured using the Nikon Eclipse upright optical microscope with 10× objective during the experiments. We used ImageJ (Version: 2.0.-rc-43/1.51h, open source image processing software, copyright: 2010-2017, National Institutes of Health, Rockville, MD, USA) to integrate the image sequences into movies to study the behavior of the cells with changing frequencies and flow rates. The images were then analyzed using ImageJ. Each image had the position information of the cells for a given frequency. The positions of the cells from the strong pDEP region to the strong nDEP region were rated from 3 to −3. For each frequency, a single cell was tracked, and its position rate noted as strong nDEP (−3), nDEP (−2), weak nDEP (−1), crossover (0), weak pDEP (1), pDEP (2) and strong pDEP (3). Then, the mean values of their positions with standard deviations were calculated for each frequency using the Prism software (GraphPad 5, GraphPad Software Inc., La Jolla, CA, USA) as demonstrated in Figure 3.

### 3.8. Statistical Analysis

The Student’s unpaired *t*-test (two-tailed) was used to assess the statistical significance of pairwise comparison for the percentage of initial and enriched live monocytes using a 3D carbon-DEP method. Three independent separation experiments were performed. *p*-values were calculated using the GraphPad Prism software. *p*-values < 0.05 were considered significant (*** *p* < 0.0001).

## 4. Results

### 4.1. Dielectrophoretic Characterization of the Live and Dead U937 Monocytes

To characterize the dielectrophoretic behavior of the viable and nonviable U937 monocytes, 20 Vpp was applied with ranging frequencies between 50 kHz to 1 MHz for 4.7 min. The images of the cells were acquired with 1-frame/second frame rate. The obtained images were manually analyzed using ImageJ as described in Section 3.7. At each frequency, the location of the cells was recorded whether the cells exhibit nDEP or pDEP. The results indicated that the viable U937 monocytes exhibited both nDEP and pDEP behaviors between the frequencies of the 50 kHz and 1 MHz, as shown in Figure 3. The crossover frequency for the live monocytes is 150 kHz. However, the dielectrophoretic forces those dead monocytes experienced were not very clear. Our experimental observations showed that dead cells exhibit either very weak nDEP forces or are not affected by the DEP forces at all (Supplementary Materials Video S1). Our simulation results were consistent with the behavior of the dead cells in the experiments (Figure 1). Therefore, we performed the live and dead monocyte separation experiments using 20 Vpp, 300 kHz.

### 4.2. Dielectrophoretic Separation of the Live and Dead U937 Monocytes

**Figure 4:**
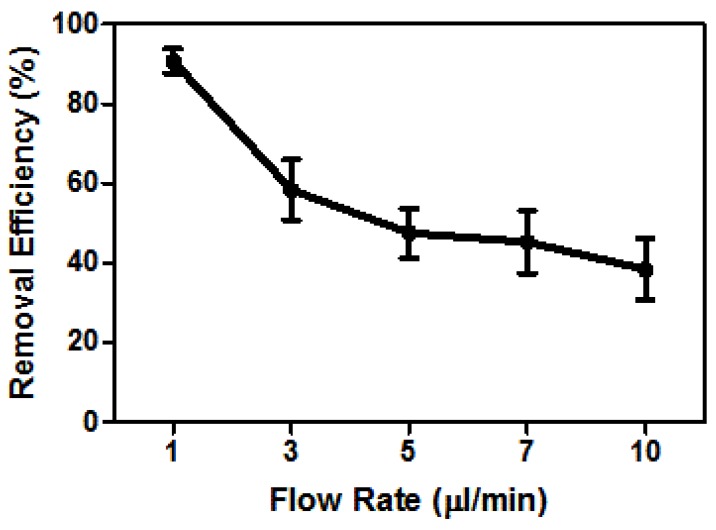
Separation efficiency with the flow rates. The live cells were captured at 20 Vpp, 300 kHz, while the dead cells were removed using 1, 3, 5, 7 and 10 μL/min flow rates. For each flow rate, three independent experiments were performed, and the error bars present the standard deviations.

As we reported above, we applied 20 Vpp, 300 kHz and conducted the experiments using a 20 μS/cm DEP buffer. To obtain high-throughput separation, we applied 1, 3, 5, 7 and 10 μL/min flow rates. We determined the flow rate dependent separation efficacy via counting the live and dead cells inside the collection tubes. Figure 4 presents the separation efficiency using different flow rates.

**Figure 5:**
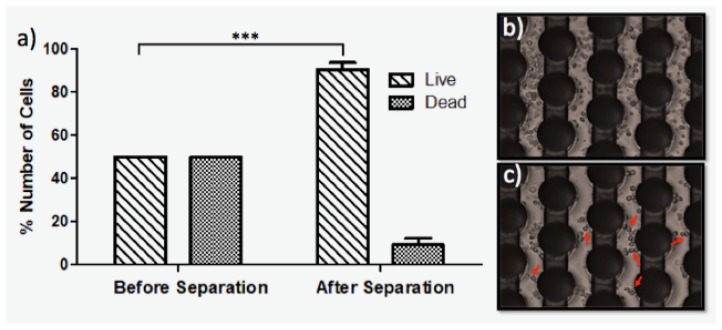
Separation of the live and dead U937 monocytes using 3D carbon-DEP chip. (**a**) the live and dead cell ratio was 1:1 in the cell culture prior to separation. Upon the separation, 90% of the dead cells were removed; (**b**) the images show the cells on the 3D carbon array before the separation and (**c**) after separation. The separation conditions: 20 Vpp, 300 kHz, 1 μL/min flow rate.

Figure 5 represents the percentage of the live and dead monocytes for pre- and post-DEP separation. We introduced 40 μL cell suspension into the 3D carbon-DEP chip and selectively concentrated the live cells at the pDEP regions using the 300 kHz, 20 Vpp electric-field for 40 min. The dead cells were removed using the 1 μL/min flow rate. Upon removal of the dead cells, the live cells remained in the pDEP inside the electrode array. When almost 90% of the dead cells were removed, the electric field was turned off, and the live cells were released and collected in the separate tubes for further analysis.

## 5. Discussion

Our experimental results show that the live monocytes experienced the dielectric forces more than the dead monocytes, as illustrated in Figure 5. The dead cells weakly responded to DEP forces due to the lack of conductivity and permittivity difference with their surrounding DEP buffer. There is a membrane polarizability difference between the live and dead cells, which provided selective removal of the dead cells from the live cells in the mixed cell population [67].

To determine the best electric field and flow conditions, we applied 20 Vpp, 50 kHz–1 MHz frequency (Figure 3) and 1–10 μL/min flow rate (Figure 4). The 20 Vpp, 300 kHz and 1 μL/min flow rate was the most effective separation condition that selectively traps live cells and overcomes the drag forces. Since the dead cells were either affected by slight nDEP forces or they did not experience the DEP force at all, they were washed from the chip with the constant fluid flow using 1 μL/min flow rate. The viability test result, Figure 5, shows that the viable monocytes were significantly enriched under these experimental conditions. The snapshot of the cells in Figure 5 demonstrated the trapped-live cells around the carbon-electrodes upon dead cell removal. Some cells polarized and, due to the dipole–dipole interaction, they formed the pearl chains (red arrows in Figure 5).

To the best of our knowledge, there is only Shafiee and his co-worker’s study related to the dielectrophoretic separation of live and dead monocytes in literature (see Table 1) [38]. They used the THP-1 monocyte cell line (Acute monocytic leukemia cell line) and applied the contactless dielectrophoresis for separation of the live THP-1 cells from the dead ones with almost 95% removal efficiency. In this approach, the major problem was cell lysis due to the required high voltages to generate DEP forces. In this study, our contribution using the 3D carbon-DEP platform is prevention of the cell damage at high voltages as 20 Vpp. In addition to our method, other DEP techniques have been applied for the separation of live and dead cells from the mixed yeast cells such as reservoir-based dielectrophoresis (Table 1) [35]. Although it was a valuable approach to increase the yield of the enriched cell population with its straightforward fabrication process compared to the 3D carbon-DEP devices, clogging of the cells in the reservoirs of this platform decreased its separation purity and efficiency. Furthermore, most of the other DEP-based methods have been implemented for the separation of live and dead bacteria separation rather than mammalian cells, as summarized in Table 1. On the other hand, most of the dielectrophoretic enrichment or isolation applications have been performed for the purification of a specific type of a cell from the other cell types, such as isolation of circulating tumor cells from other cancer cells or blood cells [68]. One of the primary motivations for these applications is when the cell size changes (*r*, Equation (1)), the dielectrophoretic force changes with its third power ($r^{3}$, Equation (1)); hence, separation of cells with different diameters becomes promising. When the subpopulations of the same type of cells with the same radius are separated, the DEP-force difference occurs only based on the real part of the Clausius–Mossotti factor (($f_{cm}$), Equation (2)), which might be a challenge for cell separation applications.

## 6. Conclusions

This work presents the dielectrophoretic characterization and separation of live and dead human U937 monocyte cells from a mixture of cell suspension using the 3D carbon-DEP method. Our separation provided a simple and efficient approach regarding the other DEP methods [69], while preventing cell lysing due to electrical field [38]. Our experimental results present significant live cell enrichment and dead cell removal for the U937 monocyte cells. This study demonstrates that 3D carbon-DEP method has a great potential for the diagnosis of certain diseases via purifying specific cell types without altering their genetic or phenotypic properties while reducing dead-cell contamination from the population in order to achieve accurate and reliable readouts from biological assays and clinical tests.

## Figures and Tables

**Table 1:** Different dielectrophoresis methods for separation of living cells from dead ones.

Cell Type	Method	Separation/Removal Efficiency	Advantages	Disadvantages
Human Leukemia (THP-1)	Contactless dielectrophoresis [38]	95% Removal Efficiency	High efficiency, inexpensive fabrication process	Lysing due to electrical field
Yeast	Reservoir-based dielectrophoresis [35]	N/A	No mechanical or electrical parts inside the microchannel	Clogging due to trapped cells
Chondrocytes	Optically-induced dielectrophoresis [68]	96.4 ± 2.2% Purity of the isolated live cells	No mechanical or electrical parts inside the microchannel	N/A
*Listeria innocua*	Interdigitated microelectrodes [32]	90% Separation Efficiency	Cells were viable for downstream analysis	N/A
*Escheria coli*	Insulator-based dielectrophoresis [31]	N/A	Differential DEP trapping was achieved	N/A
U937 Monocyte	3D carbon-dielectrophoresis	>90%	High efficiency, cells were viable for downstream analysis, no electrolysis wider electrochemical stability	N/A

## Supplementary Materials

The Supplementary Materials are available online at http://www.mdpi.com/1424-8220/17/11/2691/s1. Video S1: Live-Dead Monocyte Separation; Figure S1: The diameter of the live and dead U937 monocyte cells; Figure S2: Viability of the cells in low conductive DEP buffer; Figure S3: Viability of the cells for pre- and post-DEP exposure.

## Appendix A

For MATLAB script, visit: https://www.mathworks.com/matlabcentral/fileexchange/63572-nurdanerdem-dep.

## References

[1] Masuda T., Hayashi N., Iguchi T., Ito S., Eguchi H., Mimori K. (2016). Clinical and biological significance of circulating tumor cells in cancer. Mol. Oncol..

[2] Miyamoto D.T., Ting D.T., Toner M., Maheswaran S., Haber D.A. (2016). Single-Cell Analysis of Circulating Tumor Cells as a Window into Tumor Heterogeneity. Cold Spring Harb. Symp. Quant. Biol..

[3] Nagrath S., Sequist L.V., Maheswaran S., Bell D.W., Irimia D., Ulkus L., Smith M.R., Kwak E.L., Digumarthy S., Muzikansky A. (2007). Isolation of rare circulating tumour cells in cancer patients by microchip technology. Nature.

[4] Qin J., Alt J.R., Hunsley B.A., Williams T.L., Fernando M.R. (2014). Stabilization of circulating tumor cells in blood using a collection device with a preservative reagent. Cancer Cell Int..

[5] Riethdorf S., Fritsche H., Müller V., Rau T., Schindlbeck C., Rack B., Janni W., Coith C., Beck K., Jänicke F. (2007). Detection of circulating tumor cells in peripheral blood of patients with metastatic breast cancer: A validation study of the Cell Search system. Clin. Cancer Res..

[6] Gascoyne P.R.C., Wang X.B., Huang Y., Becker F.F. (1997). Dielectrophoretic separation of cancer cells from blood. IEEE Trans. Ind. Appl..

[7] Sueblinvong T., Ghebre R., Iizuka Y., Pambuccian S.E., Vogel R.I., Skubitz A.P.N., Bazzaro M. (2012). Establishment, characterization and downstream application of primary ovarian cancer cells derived from solid tumors. PLoS ONE.

[8] Bøyum A. (1984). [9] Separation of lymphocytes, granulocytes, and monocytes from human blood using iodinated density gradient media. Methods Enzymol..

[9] Bøyum A., Løvhaug D., Tresland L., Nordlie E.M. (1991). Separation of leucocytes: Improved cell purity by fine adjustments of gradient medium density and osmolality. Scand. J. Immunol..

[10] Thöne F., Schwanhäusser B., Becker D., Ballmaier M., Bumann D. (2007). FACS-isolation of Salmonella-infected cells with defined bacterial load from mouse spleen. J. Microbiol. Methods.

[11] Wigzell H., Sundqvist K.G., Yoshida T.O. (1972). Separation of Cells According to Surface Antigens by the Use of Antibody-Coated Columns. Fractionation of Cells Carrying Immunoglobulins and Blood Group Antigen. Scand. J. Immunol..

[12] Quirici N., Soligo D., Bossolasco P., Servida F., Lumini C., Deliliers G.L. (2002). Isolation of bone marrow mesenchymal stem cells by anti-nerve growth factor receptor antibodies. Exp. Hematol..

[13] Tomlinson M.J., Tomlinson S., Yang X.B., Kirkham J. (2013). Cell separation: Terminology and practical considerations. J. Tissue Eng..

[14] Chen J., Xue C., Zhao Y., Chen D., Wu M.H., Wang J. (2015). Microfluidic impedance flow cytometry enabling high-throughput single-cell electrical property characterization. Int. J. Mol. Sci..

[15] Inglis D.W., Riehn R., Sturm J.C., Austin R.H. (2006). Microfluidic high gradient magnetic cell separation. J. Appl. Phys..

[16] Kiermer V. (2005). FACS-on-a-chip. Nat. Methods.

[17] Rembaum A., Yen R.C.K., Kempner D.H., Ugelstad J. (1982). Cell labeling and magnetic separation by means of immunoreagents based on polyacrolein microspheres. J. Immunol. Methods.

[18] Lundahl J., Hallden G., Hallgren M., Sköld C.M., Hed J. (1995). Altered expression of CD11b/CD18 and CD62L on human monocytes after cell preparation procedures. J. Immunol. Methods.

[19] Toner M., Irimia D. (2005). Blood-on-a-chip. Annu. Rev. Biomed. Eng..

[20] Pohl H.A., Hawk I. (1966). Separation of living and dead cells by dielectrophoresis. Science.

[21] Elitas M., Martinez-Duarte R., Dhar N., McKinney J.D., Renaud P. (2014). Dielectrophoresis-based purification of antibiotic-treated bacterial subpopulations. Lab Chip.

[22] Yang J., Huang Y., Wang X., Wang X.B., Becker F.F., Gascoyne P.R.C. (1999). Dielectric properties of human leukocyte subpopulations determined by electrorotation as a cell separation criterion. Biophys. J..

[23] Pohl H.A., Crane J.S. (1971). Dielectrophoresis of cells. Biophys. J..

[24] Elitas M., Dhar N., Schneider K., Valero A., Braschler T., McKinney J.D., Renaud P. (2017). Dielectrophoresis as a single cell characterization method for bacteria. Biomed. Phys. Eng. Express.

[25] Gascoyne P.R.C., Shim S. (2014). Isolation of circulating tumor cells by dielectrophoresis. Cancers.

[26] Pethig R., Menachery A., Pells S., De Sousa P. (2010). Dielectrophoresis: A review of applications for stem cell research. BioMed Res. Int..

[27] Gascoyne P.R.C., Vykoukal J. (2002). Particle separation by dielectrophoresis. Electrophoresis.

[28] Pethig R.R. (2017). Dielectrophoresis: Theory, Methodology and Biological Applications.

[29] Carpenter E.L., Rader J., Ruden J., Rappaport E.F., Hunter K.N., Hallberg P.L., Krytska K., O’Dwyer P.J., Mosse Y.P. (2014). Dielectrophoretic Capture and Genetic Analysis of Single Neuroblastoma Tumor Cells. Front. Oncol..

[30] Tada S. Separation of live/dead cells by the use of three dimensional non-uniform AC electric field. Proceedings of the World Automation Congress (WAC).

[31] Lapizco-Encinas B.H., Simmons B.A., Cummings E.B., Fintschenko Y. (2004). Dielectrophoretic concentration and separation of live and dead bacteria in an array of insulators. Anal. Chem..

[32] Li H., Bashir R. (2002). Dielectrophoretic separation and manipulation of live and heat-treated cells of Listeria on microfabricated devices with interdigitated electrodes. Sens. Actuators B Chem..

[33] Markx G.H., Talary M.S., Pethig R. (1994). Separation of viable and non-viable yeast using dielectrophoresis. J. Biotechnol..

[34] Qian C., Huang H., Chen L., Li X., Ge Z., Chen T., Yang Z., Sun L. (2014). Dielectrophoresis for bioparticle manipulation. Int. J. Mol. Sci..

[35] Patel S., Showers D., Vedantam P., Tzeng T.R., Qian S., Xuan X. (2012). Microfluidic separation of live and dead yeast cells using reservoir-based dielectrophoresis. Biomicrofluidics.

[36] Ho C.T., Lin R.Z., Chang W.Y., Chang H.Y., Liu C.H. (2006). Rapid heterogeneous liver-cell on-chip patterning via the enhanced field-induced dielectrophoresis trap. Lab Chip.

[37] Zahn J.D. (2009). Methods in Bioengineering: Biomicrofabrication and Biomicrofluidics.

[38] Shafiee H., Sano M.B., Henslee E.A., Caldwell J.L., Davalos R.V. (2010). Selective isolation of live/dead cells using contactless dielectrophoresis (cDEP). Lab Chip.

[39] Martinez-Duarte R., Gorkin R.A., Abi-Samra K., Madou M.J. (2010). The integration of 3D carbon-electrode dielectrophoresis on a CD-like centrifugal microfluidic platform. Lab Chip.

[40] Islam M., Natu R., Larraga-Martinez M.F., Martinez-Duarte R. (2016). Enrichment of diluted cell populations from large sample volumes using 3D carbon-electrode dielectrophoresis. Biomicrofluidics.

[41] Martinez-Duarte R., Renaud P., Madou M.J. (2011). A novel approach to dielectrophoresis using carbon electrodes. Electrophoresis.

[42] Jaramillo M.d.C., Torrents E., Martínez-Duarte R., Madou M.J., Juárez A. (2010). On-line separation of bacterial cells by carbon-electrode dielectrophoresis. Electrophoresis.

[43] Martinez-Duarte R., Camacho-Alanis F., Renaud P., Ros A. (2013). Dielectrophoresis of lambda-DNA using 3D carbon electrodes. Electrophoresis.

[44] Van Furth R., Cohn Z.A. (1968). The origin and kinetics of mononuclear phagocytes. J. Exp. Med..

[45] Galkina E., Ley K. (2009). Immune and Inflammatory Mechanisms of Atherosclerosis. Annu. Rev. Immunol..

[46] Mee A.S., Berney J., Jewell D.P. (1980). Monocytes in inflammatory bowel disease: Absolute monocyte counts. J. Clin. Pathol..

[47] Lim J.K., Obara C.J., Rivollier A., Pletnev A.G., Kelsall B.L., Murphy P.M. (2011). Chemokine receptor Ccr2 is critical for monocyte accumulation and survival in West Nile virus encephalitis. J. Immunol..

[48] Dawson T.C., Beck M.A., Kuziel W.A., Henderson F., Maeda N. (2000). Contrasting effects of CCR5 and CCR2 deficiency in the pulmonary inflammatory response to influenza A virus. Am. J. Pathol..

[49] Aldridge J.R., Moseley C.E., Boltz D.A., Negovetich N.J., Reynolds C., Franks J., Brown S.A., Doherty P.C., Webster R.G., Thomas P.G. (2009). TNF/iNOS-producing dendritic cells are the necessary evil of lethal influenza virus infection. Proc. Natl. Acad. Sci. USA.

[50] Osterholzer J.J., Chen G.H., Olszewski M.A., Curtis J.L., Huffnagle G.B., Toews G.B. (2009). Accumulation of CD11b+ lung dendritic cells in response to fungal infection results from the CCR2-mediated recruitment and differentiation of Ly-6Chigh monocytes. J. Immunol..

[51] Ersland K., Wüthrich M., Klein B.S. (2010). Dynamic interplay among monocyte-derived, dermal, and resident lymph node dendritic cells during the generation of vaccine immunity to fungi. Cell Host Microbe.

[52] Tsou C.L., Peters W., Si Y., Slaymaker S., Aslanian A.M., Weisberg S.P., Mack M., Charo I.F. (2007). Critical roles for CCR2 and MCP-3 in monocyte mobilization from bone marrow and recruitment to inflammatory sites. J. Clin. Investig..

[53] Jia T., Serbina N.V., Brandl K., Zhong M.X., Leiner I.M., Charo I.F., Pamer E.G. (2008). Additive roles for MCP-1 and MCP-3 in CCR2-mediated recruitment of inflammatory monocytes during Listeria monocytogenes infection. J. Immunol..

[54] Kipnis A., Basaraba R.J., Orme I.M., Cooper A.M. (2003). Role of chemokine ligand 2 in the protective response to early murine pulmonary tuberculosis. Immunology.

[55] La Manna M.P., Orlando V., Dieli F., Di Carlo P., Cascio A., Cuzzi G., Palmieri F., Goletti D., Caccamo N. (2017). Quantitative and qualitative profiles of circulating monocytes may help identifying tuberculosis infection and disease stages. PLoS ONE.

[56] Bobdey S., Ganesh B., Mishra P., Jain A. (2017). Role of Monocyte Count and Neutrophil-to-Lymphocyte Ratio in Survival of Oral Cancer Patients. Int. Arch. Otorhinolaryngol..

[57] Niemeyer C.M., Arico M., Basso G., Biondi A., Rajnoldi A.C., Creutzig U., Haas O., Harbott J., Hasle H., Kerndrup G. (1997). Chronic myelomonocytic leukemia in childhood: A retrospective analysis of 110 cases. Blood.

[58] Sprangers S., Vries T.J.D., Everts V. (2016). Monocyte heterogeneity: Consequences for monocyte-derived immune cells. J. Immunol. Res..

[59] Pethig R. (2010). Dielectrophoresis: Status of the theory, technology, and applications. Biomicrofluidics.

[60] Morgan H., Green N.G. (2003). AC Electrokinetics: Colloids and Nanoparticles.

[61] Valero A., Braschler T., Renaud P. (2010). A unified approach to dielectric single cell analysis: Impedance and dielectrophoretic force spectroscopy. Lab Chip.

[62] Chan K.L., Gascoyne P.R.C., Becker F.F., Pethig R. (1997). Electrorotation of liposomes: Verification of dielectric multi-shell model for cells. Biochim. Biophys. Acta-Lipids Lipid Metab..

[63] Jönsson M., Welch K., Hamp S., Strømme M. (2006). Bacteria counting with impedance spectroscopy in a micro probe station. J. Phys. Chem. B.

[64] Schwan H.P. (1957). Electrical properties of tissue and cell suspensions. Adv. Biol. Med. Phys..

[65] Erdem N., Yildizhan Y., Elitasş M. (2017). A numerical approach for dielectrophoretic characterization and separation of human hematopoietic cells. Int. J. Eng. Res. Technol..

[66] Mernier G., Martinez-Duarte R., Lehal R., Radtke F., Renaud P. (2012). Very high throughput electrical cell lysis and extraction of intracellular compounds using 3D carbon electrodes in lab-on-a-chip devices. Micromachines.

[67] Prodan E., Prodan C., Miller J.H. (2008). The dielectric response of spherical live cells in suspension: An analytic solution. Biophys. J..

[68] Huang S.B., Liu S.L., Li J.T., Wu M.H. (2014). Label-free Live and Dead Cell Separation Method Using a High-Efficiency Optically-Induced Dielectrophoretic (ODEP) Force-based Microfluidic Platform. Int. J. Autom. Smart Technol..

[69] Martinez-Duarte R. (2012). Microfabrication technologies in dielectrophoresis applications—A review. Electrophoresis.

